# COVID-19 Vaccination Campaign in Cancer Patients and Healthcare Workers-Results from a French Prospective Multicenter Cohort (PAPESCO-19)

**DOI:** 10.3390/cancers14225547

**Published:** 2022-11-11

**Authors:** Valérie Seegers, Guillaume Rousseau, Ke Zhou, Audrey Blanc-Lapierre, Frédéric Bigot, Hakim Mahammedi, Aurélien Lambert, Camille Moreau-Bachelard, Mario Campone, Thierry Conroy, Frédérique Penault-Llorca, Michèle Boisdron-Celle, Martine Bellanger, Jean-Luc Raoul

**Affiliations:** 1 Department of Biostatistics, Institut de Cancérologie de l’Ouest (ICO), 44805 Saint-Herblain, France; 2 Department of Biopathology, Institut de Cancérologie de l’Ouest (ICO), 49055 Angers, France; 3 Department of Human and Social Sciences, Institut de Cancérologie de l’Ouest (ICO), 44805 Saint-Herblain, France; 4 Department of Medical Oncology, Institut de Cancérologie de l’Ouest (ICO), 49055 Angers, France; 5 Department of Medical Oncology, Centre Jean Perrin, 63011 Clermont-Ferrand, France; 6 Department of Medical Oncology, Institut de Cancérologie de Lorraine, 54511 Vandoeuvre-lès Nancy, France; 7 Department of Medical Oncology, Institut de Cancérologie de l’Ouest (ICO), 44805 Saint-Herblain, France; 8 Centre Jean Perrin, Department of Biopathology and INSERM U1240, Université Clermont Auvergne, 63011 Clermont-Ferrand, France; 9 Institut National de la Santé et de la Recherche Médicale (INSERM) U1240 Imagerie Moléculaire et Stratégies Theranostiques (IMoST), Université Clermont Auvergne, 63000 Clermont-Ferrand, France; 10 Department of Social Sciences, EHEPS School of Public Health, 35043 Rennes, France; 11 Department of Clinical Research, Institut de Cancérologie de l’Ouest (ICO), 44800 Saint-Herblain, France

**Keywords:** COVID-19, COVID-19 vaccine, cancer patients, heath care workers, serological tests, neutralizing antibody, France

## Abstract

**Simple Summary:**

Vaccination against COVID-19 was a major weapon against current epidemics. In a multicenter cohort study conducted in cancer patients (CP) and health care workers (HCW) we demonstrated that: (i) vaccination was well accepted in both cohorts; (ii) the seropositivity rate was high after the first and second injections among HCW and among CP this was only after the second injection; (iii) similar patterns in antibody response followed the second dose; in both groups, antibody levels waned 3 months after the second dose. Overall, two-dose COVID-19 vaccination was effective in both populations studied, but could not totally prevent a few vaccine breakthrough infections, owing to the continuously emerging novel variants.

**Abstract:**

In this prospective, real-life cohort study, we followed 523 cancer patients (CP) and 579 healthcare workers (HCW) from two cancer centers to evaluate the biological and clinical results of the COVID-19 vaccination campaign. Seventy percent of the CP and 90% of the HCW received an mRNA vaccine or the AZD1222 vaccine. Seropositivity was high after the first vaccine among HCW and poor among CP. The second dose resulted in almost 100% seropositivity in both cohorts. Antibody response was higher after the second injection than the first in both populations. Despite at least two doses, 8 CP (1.5%) and 14 HCW (2.4%) were infected, corresponding either to a weak level of antibody or a new strain of virus (particularly the Omicron variant of concern). Sixteen CP and three HCW were hospitalized but none of them died from COVID-19. To conclude, this study showed that two doses of COVID-19 vaccines were crucially necessary to attain sufficient seropositivity. However, the post-vaccination antibody level declines in individuals from the two cohorts and could not totally prevent new SARS-CoV-2 infections.

## 1. Introduction

The COVID-19 pandemic, caused by SARS-CoV-2, has led to major global health and social issues. In high income countries, guidelines recommended vaccination against COVID-19 for patients with cancer, who were prioritized [1,2]. However, little was known about the extent to which vaccines had been protective in this population at risk of developing a less proficient immune response [3]. Progressively, data accumulated showing that the safety of vaccines in cancer patients (CP) is similar to that of the general population and that the vast majority of CP with solid tumors under chemotherapy generated antibody responses to two doses of BNT162b2 [3,4,5,6]. We do not have reliable correlates of protection but only surrogate endpoints targeting immune response (production of neutralizing antibodies, T-cells responses) [7]. However, antibody concentration decreases over time with a decrease in immunity, prompting the proposition of a third vaccination for patients treated for solid tumors [6,7]. Griffiths and Segal report a “predicted continuum of COVID-19 vaccine efficacy in patients with cancer based on cancer type and therapy”. The protection is not far from that observed in the general population for patients with solid tumors receiving ‘active therapy,’ whilst there is no protection for lymphoma or multiple myeloma under treatment, nor for early periods of stem cell transplant [3]. However, study findings are mixed. Reduced antibody responses after COVID-19 vaccination are likely to be associated with cancer treatments. For instance, in CP with solid tumors, lower antibody response may occur in patients receiving chemotherapy, steroids, CDK4/6 inhibitors and antiPARP, while this is uncommon on endocrine therapy, tyrosine kinase inhibitors, and immune check point inhibitors [7]. Finally, the mutations affecting spike epitopes may reduce the protection induced by vaccines developed on the basis of a wild-type virus, as the CAPTURE study showed. The functional humoral response after vaccination was lower against variants of concern (VOC) (i.e., Alpha, Beta, and Delta) vs wildtype [8]. More recently, the VOC Omicron has also raised concerns [9]. Even after booster dose administration, vaccination neutralization against the Omicron variant was lower than against other VOC [9,10,11,12]. In addition, numerous factors influence vaccine effectiveness. They can be classified as host factors (such as old age, health conditions), demographic factors (such as proximity and level of circulating virus), immune factors (i.e., antibody titer and their related quality), access to vaccine, and viral variant factors [13]. Despite COVID-19 vaccine effectiveness in CP, the waning effectiveness over time (i.e., 3–6 months after the second dose) remains an issue. Studies showed that in CP, the vaccine effectiveness waned rapidly, with outcomes being COVID-19 associated hospitalization or death and breakthrough infections, especially in patients with hematological malignancies [14,15,16]. Higher breakthrough infections in CP compared to the general population may appear within months of the second dose of vaccine [15]. Furthermore, the vaccine effectiveness and protection duration may differ within community dwelling people, several studies report. Younger (i.e., below 55 years) and healthy people (w/o comorbidity) benefit from longer lasting protection against infection, after 5 months of the second dose [12,17,18,19]. Assessing the impact of COVID-19 vaccination in people and especially in CP remains challenging.

In June 2020, we initiated a real-life multicenter cohort study—the PAPESCO-19 study—, recording data from CP and health care workers (HCW) in four French Comprehensive Cancer Centers, treating only solid tumors. From this evolving series, we showed that both populations have a similar prevalence of COVID-19 infection. We investigated the symptom patterns of SARS-CoV-2 infection by highlighting the clinical importance of anosmia and emphasized the high proportion of asymptomatic cases among CP [20,21]. The aim of the current article is to analyze biological and clinical outcomes to assess the impact of vaccination campaigns among CP and HCW in a real-world setting.

## 2. Material and Methods

This section has been reported previously [20,21].

### 2.1. Study Design and Population

The PAPESCO-19 (PAtients et PErsonnels de Santé des Centres de Lutte Contre le Cancer pendant l’épidémie de COVID-19) cohort study took place in 4 French Comprehensive Cancer Centers located in Angers, Nantes, Nancy and Clermont-Ferrand. It consisted of 4 work packages: (i) serology and clinic, (ii) public health, (iii) economics, and (iv) psychology. This was a one-year study, with the first enrollment on 17 June 2020, and we closed inclusions on 16 June 2021, resulting in 2306 participants.

Participation in the study was proposed to CP aged 18 years and over, irrespective of whether they had presented symptoms since the COVID-19 outbreak, attending the centers for active treatment or for follow-up (only if treatment stopped for more than 1 year). HCW (i.e., nurses, clinicians and other cancer staff) enrolled voluntarily after being informed of the study via the cancer center’s newsletter. Cancer patients and HCWs were recruited from each of the three regions. Although both cohorts within a site would have been exposed to the same spatio-temporal spread of COVID-19, they were not designed to be comparable as the two populations were not matched on baseline characteristics such as age, sex, and comorbidities [20].

Participants were followed up every three months over a full year. All participants signed an informed consent form, and the study was conducted in accordance with the Declaration of Helsinki. The Ethics Committee (CPP-IDF VIII, Boulogne-Billancourt) approved our study (number 20.04.15) on 15 May 2020. This study was registered at ClinicalTrials.gov, Identifier: NCT04421625.

### 2.2. Data Collection

We collected the information related to CP and HCW separately. At baseline and quarterly during 12 months, hereafter labelled M0, M3, M6, M9 and M12, all participants: (i) reported the presence or not of symptoms and of documented SARS-CoV-2 infection; (ii) reported data about vaccinations received (date, name); (iii) CP reported treatment received (e.g., chemotherapy, checkpoint inhibitors, tyrosine kinase inhibitors, hormones or others); (iv) all participants had blood sampling for a rapid diagnostic test (a lateral flow immunoassay (LFIA) NG-Test^®^), and aliquots were frozen and kept for antibody detection and measurement. Of note, the PAPESCO protocol defined the quarterly visits independently of the participants ‘vaccination dates. Therefore, time between vaccination and subsequent serological sample varied from one individual to another.

Baseline demographic data, clinical details, and cancer history and treatment were recorded in electronic case report forms. Participants also reported the results of RT-PCR tests (test-confirmed SARS-CoV-2 infection), done independently of this study, in case of symptoms or possible contacts.

### 2.3. Analysis of Antibody Serum Titers

Serum from frozen aliquots was analyzed for antibodies against the spike protein (S) (Liaison^®^ SARS-CoV-2 TrimericS IgG, DiaSorin, Saluggia, Italy). These antibody assays followed WHO’s recommendations (NIBSC 20/136) for the standardization of analytical comparability. The detection threshold was 4.81 BAU/mL (Binding Antibody Units/mL), and the upper antibody titer limit was capped at 2080 BAU/mL, thus the titer differences may be higher than those recorded. We considered a serology test positive (seroconversion) when the anti-S IgG titer was above 33.8 BAU/mL, the cut-off value the manufacturer set [22]. Of note, we analyzed seroconversion rates as well as antibody responses following vaccination in participants without previous self-reported SARS-CoV-2 infection or positive serology before the first dose injection.

### 2.4. Statistical Analysis

Although the PAPESCO-19 study included participants from four cancer centers, this analysis is based on the antibody detection we completed for 1102 participants from the Nantes and Angers centers, due to some delays in collecting biological results in the two other cancer centers. In the current study, the outcomes of interest i.e., antibody response, seroconversion and SRAS-CoV-2 infection were analyzed independently in CP and in HCW. No statistical comparison was carried out between the two cohorts.

For antibody serum titers, since the values out of the aforementioned range (4.8–2080 BAU/mL) [22] were censored, we used rank distribution statistics for comparison between groups, such as age, or treatment patterns for CP.

The regular follow-up was independent from the vaccine injection, as above mentioned, therefore we used the first subsequent blood sample within the 90 days following the date of vaccination, and the delay between that date (which could be M3, M6, M9, M12 depending on the personal participant history) and the date of vaccination. We present the seroconversion rates, and the median titers (25th–75th percentile) per 2-week periods following vaccination date (D0–D14, D15–D29, D30–D44, D45–D59, D60–D74, D75–D90, with D corresponding to “Day”). We reported systematically the number of available serology titers.

To explore selection bias in the titers analysis, we compared baseline characteristics (age and sex) of participants in the PAPESCO study included in the current analysis of post-vaccination antibody titers. We performed complete case analysis without any imputation of missing data. We summarized quantitative data using mean, standard deviation (SD) for age and median, range, 25th, and 75th percentiles for titer analysis. We used Bonferroni correction for multiple *p*-value computations. We conducted all statistical analyses using R version 4.1.2 (R Core Team (2021). R: A language and environment for statistical computing. R Foundation for Statistical Computing, Vienna, Austria. URL https://www.R-project.org/, accessed on 1 November 2021).

## 3. Results

### 3.1. Participants

**Figure 1:**
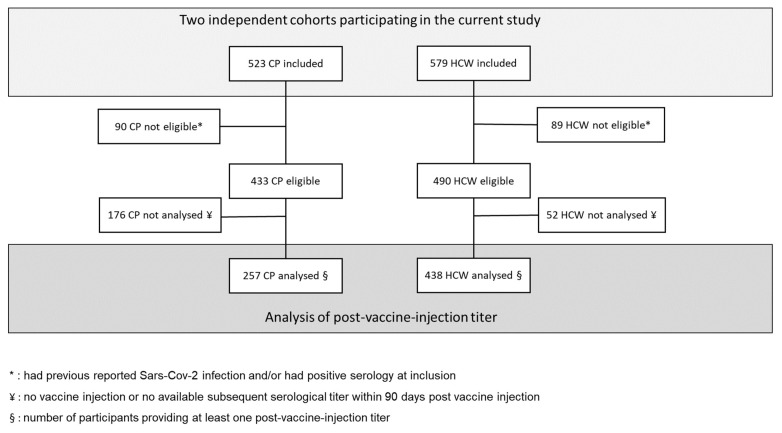
Flow-diagram for participant selection in both cohorts.

As of April 2022, complete antibody detection had been fully performed in the participants from the two centers, Angers and Nantes, composing a cohort of 1102 participants, 523 CP and 579 HCW (Figure 1).

Cancer patients had a mean age of 61 (sd = 12) years and were predominantly women (72.1%) (Table 1). CP were treated for breast (47.2%), lung (9%), gynecologic (11.5%), digestive (8.2%), urological (10.6%) or other (13.5%) cancers. Almost all (96.4%) were undergoing active cancer treatment at inclusion for localized (23.2%), locally advanced (22.6%) or metastatic (54.2%) cancers. At inclusion, their performance status was 0 in 173 cases (37.4%), 1 in 180 cases (38.9%), 2 or more in 16 cases (3.4%), and missing for 154 CP (20.3%). The last treatments, with multiple treatments possible, they received before inclusion were systemic chemotherapy (n = 307; 58.7%), targeted therapy (n = 117; 22.4%), immunotherapy (n = 76; 14.5%), hormone therapy (n = 63; 12%) radiotherapy (n = 17; 3.3%), surgery (n = 15; 2.9%) and miscellaneous (n = 3; 0.6%). During the 12-month follow-up, 287 CP (54.9%) had a change in their ongoing treatment.

Out of the 579 HCW, 482 (83.2%) were women, with a mean age of 41 (sd = 10) years.

### 3.2. COVID Vaccination

During follow-up, 147 CP (28.1%) and 68 HCW (11.8%) reported no vaccination administered (Table 1).

Baseline characteristics of unvaccinated CP were not different from those of vaccinated ones for sex, age, metastatic status, or oncological treatment. Among the unvaccinated CP, ten of them (6.8%) had a “test-confirmed” SARS-CoC-2 infection and 50 (34%) died from their cancer during the study (vs. 3.7% for vaccinated CP). Cancer patients were vaccinated (n = 376; 79.1%), mostly by mRNA BNT162b2 (n = 218; 57%) or AZD1222 (n = 39; 10.4%), less frequently by the mRNA-1273 vaccine (n = 16; 4.3%) and for more than one fourth of CP the name of the vaccine was unknown. Ninety percent of CP received a second dose of vaccine, mostly mRNA-based, within a median delay of 28 days. Fifty-two CP received a booster injection, before the end of their follow-up.

Of the 68 unvaccinated HCW, ten (14.7%) had a “test confirmed” SARS-CoV-2 infection; unvaccinated HCW were younger (mean age = 38 years, sd = 10.8) than those who were vaccinated (mean age = 41.4 years, *p* = 0.004). HCW were vaccinated (n = 511; 88.3%), by AZD1222 (n = 218; 42.7%) or mRNA vaccines (n = 273; 53.3%) for their first dose (vaccine unknown in 20); 460 had a second injection, mostly by mRNA vaccine (BNT162b2 or mRNA-1273; 87.0%) with a median delay of 26 days after a first injection in case of mRNA vaccine and 83 days after AZD1222. Eighteen HCW received a booster injection, before the end of their follow-up.

### 3.3. SARS-CoV-2 Infection in Participants

During their participation in the study, 36 CP (6.9%) and 55 HCW (9.5%) self-reported a “test-confirmed” SARS-CoV-2 infection (RT-PCR or antigen test) (Table 1). Of them, 10 CP and 10 HCW were never vaccinated.

Twenty-two participants (eight CP and 14 HCW) reported a positive test after vaccination (Supplementary Figure S1). Among the eight CP (2.1% of 376 vaccinated CP), three reported a SARS-CoV-2 infection after one dose vaccination, four following the second dose and one after full vaccination, booster included. Out of the 14 HCW (2.7% of 511 vaccinated HCW), five reported infection after one dose vaccination, six following the second dose and three after full vaccination. These 22 infections after vaccination occurred during the peak of different variant waves (i.e., nine when Alpha VOC was predominant, 8 during Delta and 5 during the most recent Omicron wave). During that last wave, one CP and three HCW tested positive for SRAS-COV2 despite a full vaccination scheme and the demonstration 2–5 weeks before of a high (above our limits) titer of neutralizing antibodies. Inversely, in seven cases (3 CP and 4 HCW) the SARS-CoV-2 infection developed in participants who had neutralizing antibodies under the limit of positivity, despite one vaccine dose in five cases and two in two cases.

Before any vaccination, 28 CP (5.4%) reported a test-confirmed SARS-CoV-2 infection. Among them, no significant association was detected according to metastatic status (*p* = 0.561), chemotherapy (*p* = 0.943), hormonotherapy (*p* = 0.135), surgery (*p* = 0.847) or targeted therapy (*p* = 0.221). No patient treated with immunotherapy reported a test-confirmed SARS-CoV-2 infection. Forty-one HCW (7.1%) reported a test-confirmed SARS-CoV-2 infection before any vaccination.

Based on serological samples collected before vaccination, 83 CP (22%) had a positive serology including 62 (74.7%) without previous “test-confirmed” SARS-CoV-2 infection, and seven CP had negative serology despite previous “test-confirmed” SARS-CoV-2 infection (Table 1). In HCW, 76 (14.9%) had positive serology before any vaccination, including 48 (63.2%) without previous reported “test-confirmed” SARS-CoV-2 infection and 13 had negative serology despite previous “test-confirmed” SARS-CoV-2 infection.

Clinically, 16 CP (3%) were hospitalized due to SARS-CoV-2 infection, with two of them in Intensive Care Unit. Regarding HCW, three (0.05%) were hospitalized. Nobody in this series died from SARS-CoV-2 infection.

### 3.4. Seroconversion Rates

**Figure 2:**
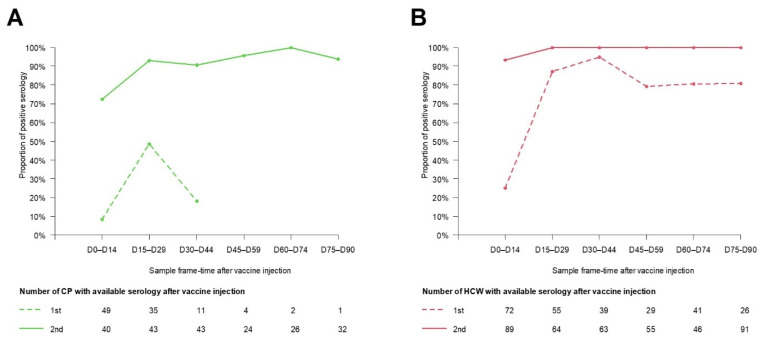
Proportion of CP (**A**) and HCW (**B**) with seroconversion in per-two-weeks after vaccination.

Among the 523 CP, 257 had available antibody titers within 90 days post vaccine injection and were included in the seroconversion rate analysis (Figure 1). Age and sex in these patients contributing to titer level analysis were not statistically different from the PAPESCO study CP. For CP, the conversion rates were 20% from D30 to D44 (Figure 2A). After the second dose injection, they attained the maximum level gradually from D45 to D59, including a small decrease beforehand (D15–D29). There was no significant difference in seroconversion rates among groups of patients on active treatment, i.e., chemotherapy, immunotherapy or hormone therapy.

From the 579 HCW, 438 had available antibody titers within 90 days post vaccine injection and were included in the seroconversion rate analysis (Figure 1). They were significantly slightly older than HCW not included in the titer analysis (mean: 39.3, sd = 10.1, *p* = 0.022). For HCW, the conversion rates reached 94.9% from D30 to D44. After the second dose injection, the rates plateaued at 100% from D15 onward (Figure 2B).

### 3.5. Antibody Response following Vaccination

Among CP, the median values were below the detection threshold in the first 2 weeks after the first vaccine injection, followed by a mild increase. After the second vaccine injection, the values observed between D0 and D14 were higher than those after the first vaccine injection, and they rose thereafter before a slow decrease beginning at D45 and lasting until the D75–D90 period (Table 2). We found no statistically significant higher titer values in those under chemotherapy compared to immunotherapy. Differences were not statistically significant in relation to CP’s age either.

Among HCW without previous self-reported SARS-CoV-2 infection or positive serology before the first vaccine injection, the median values (25th–75th percentiles) of neutralizing antibodies were <4.81 BAU/mL, i.e., below the detection range, in the first 2 weeks, then increased to a peak reached between D15 and D29 before a slow decrease (Table 3). After the second vaccine injection, titers increased very quickly and the values after this second dose were immediately higher than observed at any time after the first one and higher than our upper limit (>2080) until D60, from which they decreased.

## 4. Discussion

In France, seven waves of SARS-CoV-2 infection were observed: March–May 2020 (wild-type virus), September–November 2020, March–April 2021 (variant of concern Alpha), July–August 2021 (variant Delta), November 2021–February 2022 (variant Omicron), March–April 2022 and summer 2022. Vaccination began in the last days of December 2020 with an mRNA vaccine (BNT162b2) for CP and HCW over 50 years old, and Astra-Zeneca for younger HCW. Very quickly the mRNA BNT162b2 was used predominantly. In July 2022, 54.5 million French citizens had received one injection, 53.6 million two injections and 40.4 million had an additional booster (https://datavaccin-covid.ameli.fr, accessed on 24 July 2022).

The results presented in this manuscript correspond to patients included in two cancer centers from 17 June 2020 to 16 June 2021 and followed for 1 year. They represent about half of the PAPEPSCO cohort of 2306 participants, corresponding to all patients included in two centers, 523 CP and 579 HCW. Overall, more than 90% of HCW and 75% of CP were included before October 2020, meaning that their follow-up was closed in October 2021.

During that period, in our series 71.9% of CP and 88.3% of HCW had had at least one vaccine injection. In July 2022, in France, more than 91% of CP had received two injections and more than 85% had received a booster injection (https://datavaccin-covid.ameli.fr, accessed on 24 July 2022). In our series figures are lower because inclusions began more than 6 months before implementation of vaccination, ten CP had a “test confirmed” SARS-CoV-2 infection (initially not considered as an indication for vaccination) and 50 of the unvaccinated patients died from their cancer before the end of the one-year follow-up. No baseline characteristics of the unvaccinated CP differed from those observed in the vaccinated group. In France, vaccination of HCW was mandatory as of mid-October 2021, i.e., after the end of the follow-up for most of HCW enrolled in the cohort. It is then likely that some of the 68 unvaccinated HCW were vaccinated thereafter. Moreover, ten of them had a “test confirmed” SARS-CoV-2 infection early.

During their follow-up, 6.9% of CP and 9.5% of HCW declared a “test confirmed” SARS-CoV-2 infection; most infections were acquired before vaccination. While only a few individuals had SARS-CoV-2 infection after the first injection, others previously received two or three vaccine doses resulting in a very high titer of neutralizing antibodies a few weeks before infection. This may be related to the Omicron VOC as these cases were observed in 2022. In Israel, among 1497 fully vaccinated HCW, 39 SARS-CoV-2 infections (RT-PCR+) were observed; in the peri-infection period these HCW had lower neutralizing titers than matched uninfected controls; moreover in 85% of the cases, the Alpha variant was responsible for the infection [23]. In our study, among the 14 individuals (out of 22) who developed a demonstrated SARS-CoV-2 infection despite being vaccinated at least two doses, six (two CPs and four HCW) declared SARS-CoV-2 infection soon after demonstration of very high neutralizing antibodies on blood tests, and five were infected in early 2022 (Omicron wave). In contrast, in five individuals (three CP, two HCW) neutralizing antibody titers before infection were under the limit of detection. Thus, in our experience, infection despite at least two vaccine doses is possible and may be related to either a poor humoral response or to the VOC. This was also clearly shown in Israeli and Dutch experiences [6,23]. A recent review confirmed that, in patients with breakthrough SARS-CoV-2 infections, levels of antibodies were reduced or undetectable [7]. In addition, vaccine effectiveness might wane more rapidly for cancer patients [14,15,16]. But a dramatic reduction in neutralization of Omicron by vaccine-induced antibodies has been observed compared to the wild-type strain [10,11].

From this PAPESCO study, in a first analysis, we evaluated in 878 CP and 940 HCW (including the participants included in the present study) the prevalence of SARS-CoV-2 infection using a rapid diagnostic test the and demonstrated that 41% of CPs and only 9% of HCWs with a positive rapid test never developed symptoms [20,21]. In the current analysis based on serological tests, before any vaccination, 22% of CP had a positive serology but three quarters did not have any clinical signs leading to a RT-PCR. Surprisingly in HCW, there were 14.9% of positive serologies and more than two thirds not explored by RT-PCR. Inversely, we observed some CP and HCW who had a demonstrated SARS-CoV-2 infection but with negative serology. This may be related to the natural decrease in humoral response. We observed, particularly among HCW, that the titer of neutralizing antibodies after the first vaccine dose was high in the second–fourth week before decreasing by three quarters at 10 weeks. Antibody levels wane over time and the average time from seroconversion to seroreversion was estimated at 3–4 months [24]. This translates into a gradual decrease in effectiveness after a second dose, with a peak of effectiveness one month after vaccination and a major decrease after the fourth month [25]. A large series evaluated prospectively the waning humoral response to BNT162b2 vaccine over 6 months and demonstrated that the level of IgG antibodies decreased at a constant rate, whereas the neutralizing antibody level decreased rapidly for the first 3 months with a slow decrease thereafter [26]. This decrease in humoral response was marked among men, people over 65 years of age, and in people with immunosuppression [26]. There is accumulating evidence showing variation in COVID-19-vaccine protection along with waning vaccine effectiveness against infection, recent studies report based on large community setting in the UK [12,17,18,19]. Furthermore, the Omicron variant is less sensitive to current vaccines and AZD122 had no effect, while the effectiveness of two BNT162b2 doses was 65.5% at 2 weeks, dropping under 10% at 6 months. Maximal immunogenicity of mRNA vaccines is achieved after three doses and only restored by a fourth dose, without clear efficacy in HCW [27].

After the first vaccine injection, seroconversion rates were very high in HCW (above 85% after week 2) but were low in CP (less than 50% between weeks 2 and 4 and down to less than 20% thereafter). However, after the second vaccine dose, in both populations the seroconversion rate reached 100%. Similarly, in London, 151 patients with cancer (95 with solid tumors and 56 with hematologic cancers) and 54 healthy controls received BNT162b2 vaccine 21 days apart; 21 days after the first injection 94% of healthy controls, 38% of patients with solid tumors and 18% of those with hematologic cancers were seropositive. However 2 weeks after a second dose, 100% of controls (12/12), 95% (18/19) of patients with solid tumors and 3/5 patients with hematologic cancers were seropositive [5]. The CAPTURE study evaluated 585 patients with cancer following administration of two doses of BNT162b2 or AZD1222 vaccines administered 12 weeks apart. After two doses, the seroconversion rates were 85% and 59%, respectively, in patients with solid or hematologic malignancies [8]. In our experience with CP (solid tumors), the seroconversion rate does not depend on the anticancer treatment, but we did not include hematologic cancers. In Israel, 102 adults with solid tumors under active chemotherapy and 78 controls (same age) received their second dose of the BNT162b2 vaccine at least 12 days before enrollment; at this moment 100% of controls and 90% of cancer patients were seropositive but the median titer was significantly lower in CP than in the controls [4]. In our population, 2 weeks after the second injection the seroconversion rate was close to 100% in both cohorts but the median titer of neutralizing antibodies remained at 1600 BAU/mL in CP. It thus seems that at least two doses of vaccine are necessary to reach good vaccination efficacy, but these results also support the short interval between the two first doses. In a systematic review and meta-analysis of vaccination in CP (89 reports, 30,183 subjects) the overall seropositive rate within the first month after complete prime vaccination was 80%, 60% in patients with hematologic malignancies and 94% in patients with solid malignancies. The overall humoral response was 79%, 2 months after complete vaccination [28]. They also demonstrated that seropositivity remains stable at 2 months after primo-vaccination.

Neutralizing antibody titers are also a major parameter that needs to be assessed. In our population, humoral response to vaccine occurred early (after 2 weeks in both groups) and the increase after the second dose was clearly seen within 2 weeks. After the second dose, the decrease in the titers was low and titers observed at 3 months were similar to those observed one month before and far higher than those observed at any moment after the first dose. But titers were low in CP and patients under immunotherapy tended to have, after two doses, lower titers than those under systemic chemotherapy. In the literature, the results are very similar. In a Greek experience, in CP treated with checkpoint inhibitors and vaccinated with the BNT162b2 or AZD1222 vaccines, despite a poor humoral response after the first injection, the seroconversion rate increased after full vaccination up to 73.5%. A steady decline in neutralizing antibodies was then noted over 6 months, corrected by a third booster dose [29]. In another series of 112 CPs, 44 receiving immune checkpoint inhibitors, levels of anti-SARS-CoV-2 antibodies decreased significantly, and this decrease was more pronounced in patients receiving immune check point inhibitors [30].

Post second dose, in the CAPTURE study, the proportion of patients with detectable neutralizing antibody (NAb) titers against SARS-CoV-2 wild-type was higher than versus VOC; median titers were lower in patients with hematological malignancies vs solid cancers. In the CAPTURE study, lack of previous SARS-CoV-2 infection, Astra-Zeneca vaccine, older age, and hematological malignancies were associated with reduced NAb titers to wild-type but also to VOC post first and second dose [8]. The authors compared Nab response against the Delta VOC induced by vaccination between cancer patients in their study and healthy participants in the Legacy study, vaccinated with either the Astra-Zeneca vaccine [31] or BNT162b2 vaccine [32]. In individuals vaccinated with BNT162b2, fewer (but not significantly) cancer patients had detectable Nab titers post-second dose than individuals without cancer.

Cellular response is also of importance and in the CAPTURE study, in cancer patients, a T-cell response could be observed in patients without detectable Nabs and for example from 12 patients with hematologic malignancies without detectable Nabs, 11 had a T-cell response [8].

The strengths of our study include a large, prospectively recruited cohort of CP and HCW from different French centers corresponding to a real-world, unbiased series (50 CP died during follow-up) with a good one year of follow-up. Serological analyses were performed late, after the end of the inclusion period on frozen specimen in order to use a “validated” test. We acknowledge some limitations in our data and in our methodology. We did not determine the type of variant when a RT-PCR test was carried out, and “test-determined” SARS-CoV-2 infections were self-reported by participants and could not be checked. This may have introduced information bias and led to underestimating SARS-CoV-2 infections. In addition, self-reporting the date of vaccination may have affected estimate precision in post-vaccination antibody titer analyses by 2 week periods. However, there is no a priori reason to suspect systematic biases in these analyses. Besides, we excluded participants who were not able to report their vaccination dates accurately from antibody titer analyses, likely resulting in selecting healthier patients. Due to the selection bias, HCW contributing to the analysis might be less representative of the whole PAPESCO cohort of HCW. Despite the high number of participants included, blood tests were done quarterly and vaccination when possible; this could not preclude some issues in the follow-up of CP and HCW. Finally, our analysis of breakthrough infections remains descriptive due to the few cases observed in our study.

## 5. Conclusions

To conclude, our experience is in accordance with others that show that SARS-CoV-2 vaccines give more benefits than risks, even in patients with cancer, and that after a second dose of BNT162b2 most CP developed antibodies. Unfortunately, despite good humoral response, less intense in CP than in HCW, likely less important in patients on immunotherapy than on chemotherapy, the appearance of variants of concern, and particularly the Omicron variant, is a major issue. A third and fourth vaccine injection boost the immune response but will certainly not be enough. An Omicron-adapted vaccine recently in use and, in association with the usual precautions, may help to overcome breakthrough infection.

## Figures and Tables

**Table 1:** Participant characteristics. CP: cancer patients; HCW: health care workers; F: female; ICU: intensive care unit.

	CP n = 523	HC n = 579
Age: mean (sd)	61 (12)	41 (10)
Sex: F (%)	377 (72.1%)	482 (83.2%)
Vaccination reported during follow-up		
No	147 (28.1%)	68 (11.8%)
1 injection	34 (6.5%)	50 (8.8%)
2	290 (55.4%)	444 (76.7%)
3	52 (9.9%)	17 (2.9%)
Confirmed SARS-CoV-2 infection (self-reported)	36 (6.9%)	55 (9.5%)
never vaccinated	10	10
before vaccination	18	31
after vaccination	8	14
Positive Serology * without or before vaccination	83 (22%)	76 (14.9%)
Previous test-confirmed infection	21	28
Negative Serology * before vaccination	293 (78%)	503 (86.9%)
Previous test-confirmed infection	7	13
Hospitalization due to COVID	16 (3%)	3 (%)
ICU	2	0
Death (considered as related to COVID)	0	0

* Titer > 33.8 BAU/mL corresponds to positive serology and titer ≤ 33.8 BAU/mL corresponds to negative serology.

**Table 2:** Results of antibody response following vaccination in Cancer Patients: titer of neutralizing antibodies expressed as median (25th, 75th percentiles) (in BAU/mL) according to participant characteristics (at baseline) and date of sample collection regarding the vaccine injection.

	D0–D14	D15–D29	D30–D44	D45–D59	D60–D74	D75–D90
**All CP**						
1st injection	<4.81 (<4.81, <4.81)/49	32 (<4.81, 187)/35	18 (4, 30)/11	97 (62, 341)/4	n = 2	n = 1
2nd injection	138 (32, 450)/40	1600 (505, >2080)/43	1560 (485, >2080)/43	1270 (364, 2070)/24	728 (358, 1538)/26	730 (423, 1383)/32
**CP with chemotherapy**						
1st injection	<4.81 (<4.81, <4.81)/26	23 (<4.81, 81)/18	22 (17, 30)/7	n = 1	n = 1	n = 1
2nd injection	103 (26, 520)/28	1910 (703, >2080)/29	1835 (656, >2080)/26	1670 (760, 2015)/11	782 (315, 1840)/15	810 (494, 1255)/19
**CP with immunotherapy**						
1st injection	<4.81 (<4.81, <4.81)/7	9.8 (<4.81, 77)/6	n = 2	n = 1	No serology	No serology
2nd injection	79 (8, 172)/4	528 (296, 1371.5)/6	2030 (716, >2080)/7	980 (472, 1810)/7	142 (93, 300)/3	817 (481, 1290)/6
**Women CP**						
1st injection	<4.81 (<4.81, <4.81)/31	6.8 (<4.81, 264.5)/26	16.6 (6.4, 29.7)/8	n = 2	n = 2	n = 1
2nd injection	174 (46.2, 565)/24	1890 (716.8, >2080)/34	1860 (634, >2080)/30	1270 (467, 1992.5)/20	776 (421, 1667.5)/20	1010 (492, 1430)/21
**Men CP**						
1st injection	<4.81 (<4.81, <4.81)/18	<4.81 (<4.81, 13.1)/9	21.7 (10.8, 47.9)/3	n = 2	No serology	No serology
2nd injection	93.9 (4.9, 215.8)/16	455 (380, 1190)/9	814 (386, 1170)/13	1251.5 (307.8, 2100)/4	526 (219.8, 1027.2)/6	576 (308, 834.5)/11
**CP aged 60 y and over**						
1st injection	<4.81 (<4.81, <4.81)/24	11.9 (<4.81, 77.6)/24	18 (8.6, 29.3)/5	n = 2	n = 1	No serology
2nd injection	101.5 (9.6, 258.5)/24	1500 (464.5, >2080)/22	1140 (303, >2080)/23	989 (123.1, 1937.5)/14	599 (304, 1170)/17	576 (241, 1015)/19
**CP younger than 60 y**						
1st injection	<4.81 (<4.81, <4.81)/25	85.6 (49.2, 252)/11	18.5 (3.8, 28.4)/6	n = 2	n = 1	n = 1
2nd injection	230.5 (46.2, 616.2)/16	1910 (550, >2080)/21	1800 (778.8, >2080)/20	1745 (660.8, >2080)/10	1240 (685, 2050)/9	1210 (810, 1430)/13

CP: cancer patients; n: number of available serological titers; CT: chemotherapy; IT: immunotherapy.

**Table 3:** Results of antibody response following vaccination in Health Care Workers: titer of neutralizing antibodies expressed as median (25th, 75th percentiles) (in BAU/mL) according to participant characteristics (at baseline) and date of sample collection regarding the vaccine injection.

	D0–D14	D15–D29	D30–D44	D45–D59	D60–D74	D75–D90
**All HCW**						
1st injection	<4.81 (<4.81, 31)/72	236 (70, 573)/55	168 (75, 267)/39	84 (36, 133)/29	77.8 (37.5, 122)/41	74 (39, 117)/26
2nd injection	1950 (219, >2080)/89	>2080 (2038, >2080)/64	>2080 (1880, >2080)/63	>2080 (1570, >2080)/55	1475 (889, >2080)/46	1400 (876, 1925)/91
**Women HCW**						
1st injection	<4.81 (<4.81, 26.5)/59	236 (53.6, 583.5)/47	168 (87.9, 358)/33	95.4 (36.7, 133)/25	70.6 (37.4, 131)/35	79.4 (55.1, 136)/22
2nd injection	1655 (175.8, >2080)/76	>2080 (2037, >2080)/52	>2080 (1880, >2080)/52	>2080 (1585, >2080)/44	1520 (905, >2080)/40	1450 (884, 1960)/76
**Men HCW**						
1st injection	6.9 (<4.81, 137)/13	259.5 (159.2, 418.2)/8	126.8 (61.4, 185.5)/6	42 (31, 82.9)/4	91.1 (88.8, 100.4)/6	32.4 (30.8, 33.6)/4
2nd injection	>2080 (776, >2080)/13	>2080 (1973, >2080)/12	2030 (1785, >2080)/11	1780 (1475, >2080)/11	1250 (929, 1922.5)/6	1060 (801.5, 1690)/15
**HCW aged 40 y and over**						
1st injection	<4.81 (<4.81, 31.7)/38	236 (93, 449)/33	183 (112.7, 243.2)/20	38 (34.2, 95.4)/9	74.2 (43.9, 143.8)/14	73.5 (41.1, 80.6)/9
2nd injection	1800 (269.2, >2080)/44	>2080 (1620, >2080)/37	>2080 (1620, >2080)/41	1740 (1420, >2080)/27	976 (577, 1605)/19	1150 (763, 1800)/41
**HCW younger than 40 y**						
1st injection	<4.81 (<4.81, 25.1)/34	370.5 (52.4, 931.8)/22	143 (59, 310)/19	106 (40.5, 187.2)/20	82.1 (37.4, 101.5)/27	78.3 (36.9, 139)/17
2nd injection	1950 (175, >2080)/45	>2080 (>2080, >2080)/27	>2080 (2048, >2080)/22	>2080 (1710, >2080)/28	1890 (1435, >2080)/27	1550 (986.8, >2080)/50

HCW: health care workers, n: number of available serological titers.

## Data Availability

The data that support the findings of this study are available on request from one of V.S.

## Supplementary Materials

The following supporting information can be downloaded at: https://www.mdpi.com/article/10.3390/cancers14225547/s1, Figure S1: Available serological sample titers in 8 CP and 14 HCW with SARS-CoV-2 infection after vaccination during their follow-up.
