# Supplementary figures and images for: COVID-19 Vaccination Campaign in Cancer Patients and Healthcare Workers-Results from a French Prospective Multicenter Cohort (PAPESCO-19)
Source: Cancers (Basel). 2022 Nov 11;14(22):5547. doi: 10.3390/cancers14225547 (PMC9688516; doi:10.3390/cancers14225547)

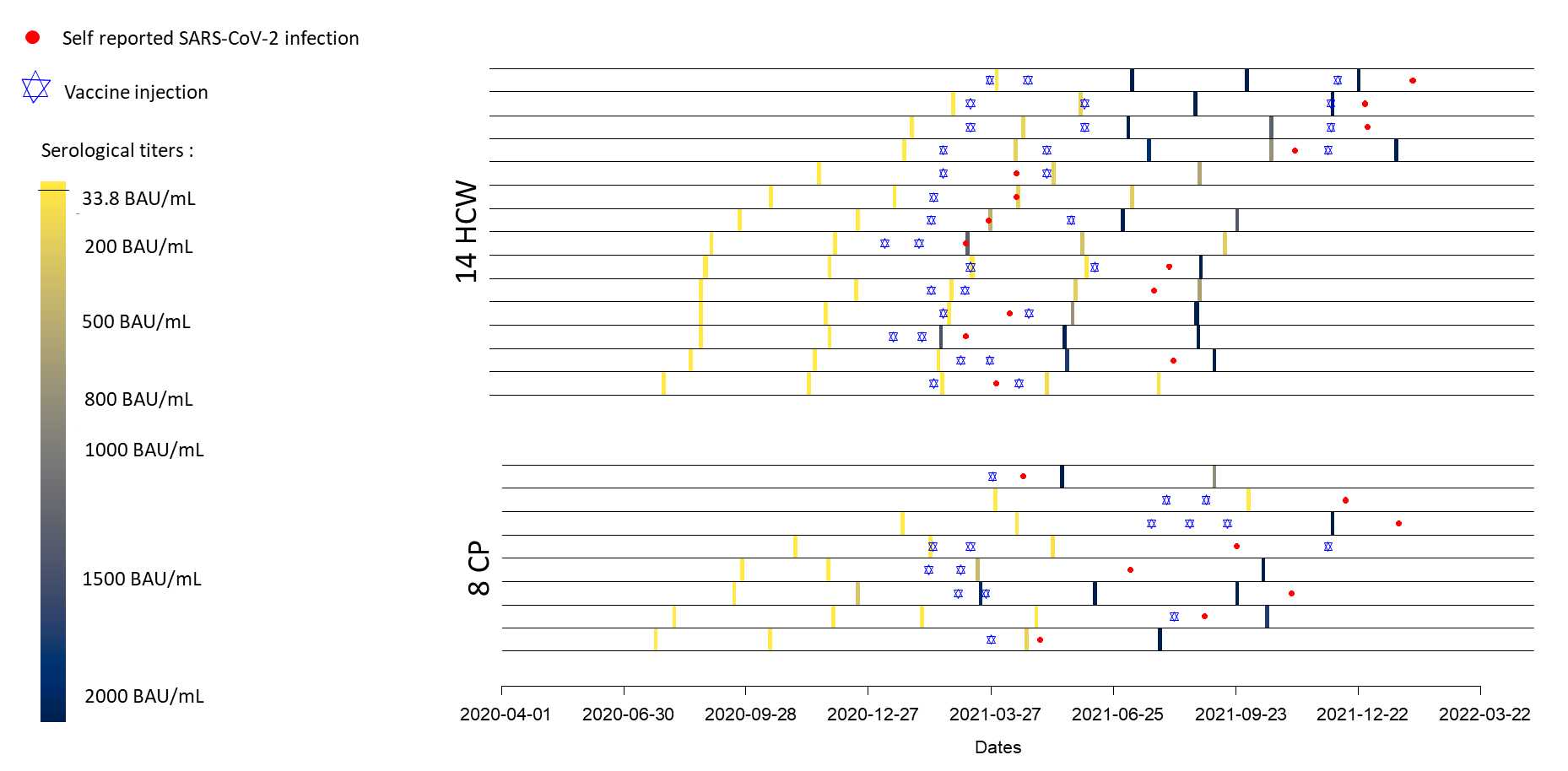

Supplement: Supplementary file 1 [file cancers-14-05547-s001.zip › supplementary figure S1.gimp.png]
